# Seasonal Changes in Chemical Profile and Antioxidant Activity of *Padina pavonica* Extracts and Their Application in the Development of Bioactive Chitosan/PLA Bilayer Film

**DOI:** 10.3390/foods11233847

**Published:** 2022-11-28

**Authors:** Martina Čagalj, Lidija Fras Zemljič, Tjaša Kraševac Glaser, Eva Mežnar, Meta Sterniša, Sonja Smole Možina, María del Carmen Razola-Díaz, Vida Šimat

**Affiliations:** 1 University Department of Marine Studies, University of Split, Ruđera Boškovića 37, 21000 Split, Croatia; 2 Laboratory for Characterization and Processing of Polymers, Faculty of Mechanical Engineering, University of Maribor, Smetanova 17, 2000 Maribor, Slovenia; 3 Department of Food Science and Technology, Biotechnical Faculty, University of Ljubljana, Jamnikarjeva 101, 1000 Ljubljana, Slovenia; 4 Department of Nutrition and Food Science, Campus of Cartuja, University of Granada, 18071 Granada, Spain

**Keywords:** functional PLA films, seaweed and chitosan bilayer, sustainable natural antioxidants, microwave-assisted extraction

## Abstract

Seaweeds are a potentially sustainable source of natural antioxidants that can be used in the food industry and possibly for the development of new sustainable packaging materials with the ability to extend the shelf-life of foods and reduce oxidation. With this in mind, the seasonal variations in the chemical composition and antioxidant activity of brown seaweed (*Padina pavonica*) extracts were investigated. The highest total phenolic content (TPC) and antioxidant activity (measured by ferric reducing/antioxidant power (FRAP), 2,2-diphenyl-1-picrylhydrazyl (DPPH) radical scavenging, and oxygen radical absorbance capacity (ORAC)) were found for *P. pavonica* June extract. The TPC of 26.69 ± 1.86 mg gallic acid equivalent/g, FRAP of 352.82 ± 15.41 µmole Trolox equivalent (TE)/L, DPPH of 52.51 ± 2.81% inhibition, and ORAC of 76.45 ± 1.47 µmole TE/L were detected. Therefore, this extract was chosen for the development of bioactive PLA bilayer film, along with chitosan. Primary or quaternary chitosan was used as the first layer on polylactic acid (PLA) films. A suspension of chitosan particles with entrapped *P. pavonica* extract was used as the second layer. X-ray photoelectron spectroscopy confirmed the presence of layers on the material surface. The highest recorded antioxidant activity of the newly developed films was 63.82% inhibition. The developed functional films exhibited antifogging and antioxidant properties, showing the potential for application in the food industry.

## 1. Introduction

Seaweed *Padina pavonica* belongs to the genus *Padina*, family Dictyotaceae, order Dictyotales, and class Phaeophyceae. Currently, there are 60 taxonomically recognized species names in the genus *Padina*. The species of this genus are widely distributed from tropical to temperate seas [1]. Due to its availability and proven biological potential [2,3,4], *P. pavonica* is a good choice for the production of biologically active extracts that can be used in many food industry processes, including packaging. Moreover, the chemical composition and biological activity of seaweeds vary depending on the season, growth phase, and other environmental factors [5,6]. In this regard, *P. pavonica* extracts were found to have the strongest antibacterial activity against human and fish pathogens during the period from April to September [7]. The highest antitumor activity of fucoidans was found in samples collected in June [8]. Since there are no reports of seasonal changes in the chemical profiles or antioxidant activity of this species, it is important to determine the best time to harvest seaweeds and prepare extracts for further exploitation. It is also important to choose the right extraction method to extract higher amounts of bioactive compounds and obtain stronger bioactivity [3]. 

In the food industry, new sustainable packaging materials should be developed and integrated with new packaging solutions to reduce the environmental footprint (both in terms of biodegradability and the origin of raw materials for packaging production). Apart from environmental aspects, new packaging solutions should also be functional and provide direct benefits to consumers by extending food shelf-life, ensuring food safety, and monitoring food quality [9]. New bio-based coatings are the trend for implementing active packaging concepts [10]. Active coated films with antioxidant properties interact with food, change its conditions, and control its quality [11].

On the other hand, biopolymers used for the development of new packaging solutions should be economical, abundant, and come from renewable sources. Polylactic acid (PLA), a thermoplastic polyester that is biodegradable, is obtained from renewable sources. PLA is one of the most widely used bioplastics due to its mechanical and physical properties. To achieve the active functionality and biodegradability of food packaging, the most commonly used biopolymers are polysaccharides, such as chitosan [12,13,14,15]. Chitosan, which is derived from the waste of marine crustaceans (shrimp, shellfish, crabs, and lobsters), is abundant, natural, and biodegradable [16]. However, chitosan itself has some limitations, such as low antioxidant capacity. Therefore, it has been shown that colloidal complexes based on the combination of chitosan and other substances, such as surfactants, polyphenols, etc., can overcome these drawbacks [17,18].

The variations in the chemical profile and biological activity of *P. pavonica* during seasonal growth, as well as the use of seaweed extracts for the development of functional PLA films alone or in combination with chitosan have not yet been investigated. In our previous research, the general concept of packaging materials with layer-by-layer colloidal chitosan-extract-polyphenol coatings for polypropylene and polyethylene was developed [18,19,20]. Therefore, the aim of this study was: (i) to determine the changes in total phenolic content (TPC) and chemical profile of *P. pavonica* during seasonal growth (from May to September); (ii) to determine the antioxidant activity of *P. pavonica* extracts; (iii) to develop and physicochemically characterize PLA films with bilayers of primary or quaternary chitosan in combination with *P. pavonica* extract—for the first time in this formulation; and (iv) to determine the antioxidant activity of the developed active packaging.

## 2. Materials and Methods

### 2.1. Collection, Extraction, and Compound Analyses

**Figure 1:**
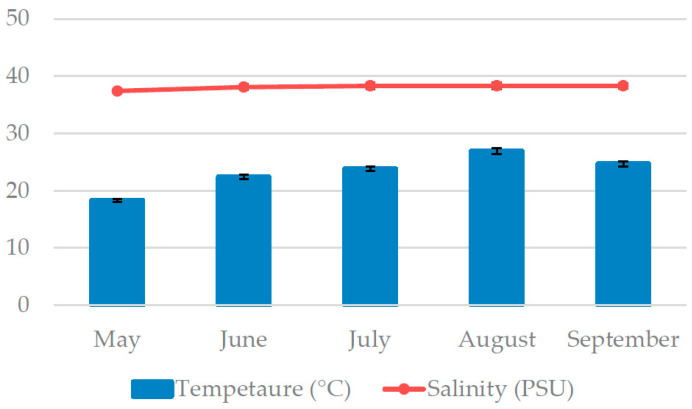
Sea temperature and salinity measured during harvesting of *P. pavonica*.

*Padina pavonica* was sampled in the Adriatic Sea off the southern coast of the island Čiovo (43.493389° N, 16.272505° E) from May to September 2020. The depth range was from 20 to 80 cm. A YSI Pro2030 probe (Yellow Springs, OH, USA) was used for the sea temperature and salinity measurements during the sampling (Figure 1). Seaweed samples were washed with tap water and freeze-dried (FreeZone 2.5, Labconco, Kansas City, MO, USA).

Freeze-dried seaweed samples were pulverized and extracted based on our previous research [3]. The seaweed powder was mixed with 50% ethanol (Gram-Mol, Zagreb, Croatia) in a 1:10 (*w*/*v*) seaweed-to-solvent ratio. Microwave-assisted extraction (MAE) was performed in an advanced microwave extraction system (ETHOS X, Milestone Srl, Sorisole, Italy) at 200 W and 60 °C for 15 min. After the extraction, samples were centrifuged for 8 min at 5000 rpm and room temperature and filtered. The ethanol was evaporated at 50 °C in a rotary evaporator and the remaining water extract was freeze-dried.

Total phenolic content (TPC) was determined by the Folin–Ciocalteu method [21], previously described by Čagalj et al. [5]. In brief, 125 μL of Folin–Ciocalteu reagent and 1.5 mL of distilled water were combined with 25 μL of the sample. After stirring the mixture for a minute, 375 μL of a 20% sodium carbonate solution and 475 μL of distilled water were added. Samples were kept at room temperature in the dark for two hours. Absorbance was measured at 765 nm. The results were expressed as mg gallic acid equivalents (GAE)/g of freeze-dried extract.

The analysis of compounds from *P. pavonica* was performed by dissolving 3 mg of freeze-dried extract in 1 mL of methanol/water (*v/v*) (Merck KGaA, Darmstadt, Germany) using the ACQUITY Ultra Performance LC system equipped with a photodiode array detector with binary solvent manager (Waters Corporation, Milford, MA, USA) series with a mass detector Q/TOF micromass spectrometer (Waters) with electrospray ionization (ESI) source operating in negative mode (UPLC-PDA-ESI-QTOF). The conditions were as follows: capillary voltage, 2300 kV; source temperature, 100 °C; cone gas flow, 40 L/h; desolvation temperature, 500 °C; desolvation gas flow, 11,000 L/h; and scan range, *m/z* 50–1500. Individual compounds’ separation was performed using an ACQUITY UPLC BEH Shield RP18 column (1.7 µm, 2.1 mm × 100 mm; Waters Corporation, Milford, MA, USA) at 40 ◦C. Water containing 1% acetic acid (A) and acetonitrile (B) (Merck KGaA, Darmstadt, Germany) was used for the elution gradient test and applied as follows: 0 min, 1% B; 2.3 min, 1% B; 4.4 min, 7% B; 8.1 min, 14% B; 12.2 min, 24% B; 16 min, 40% B; 18.3 min, 100% B, 21 min, 100% B; 22.4 min, 1% B; 25 min, 1% B. The injected sample volume was 2 µL and the used flow rate was 0.6 mL/min. The wavelength of 280 nm was used to monitor the compounds. MassLynx 4.1 software (Waters Corporation, Milford, MA, USA) was used for the integration and data elaboration [5,22].

### 2.2. Collection, Extraction, and Compound Analyses

The antioxidant activity of *P. pavonica* extracts was measured by using three different methods with different mechanisms of action. Ferric-reducing/antioxidant power (FRAP) method is based on electron transfer, while 2,2-diphenyl-1-picrylhydrazyl (DPPH) radical scavenging ability and oxygen radical absorbance capacity (ORAC) methods are based on hydrogen atom transfer [23]. The freeze-dried extracts were dissolved in 50% ethanol prior to the antioxidant activity assays.

The used FRAP method was previously described by Benzie and Strain [24] with modifications described in Čagalj et al. [5]. In brief, the microplate wells were filled with 300 μL of FRAP reagent solution and the absorbance at 592 nm was measured. Four minutes after adding 10 μL of the sample to the microplate wells, the absorbance change was measured. Measured absorbances were compared with the readings obtained for the standard Trolox solutions. The FRAP results were expressed as micromoles of Trolox equivalents/liter of extract (µmole TE/L).

The ability to scavenge DPPH radicals was measured using the method previously described by Milat et al. [25]. DPPH radical solution (290 μL) was pipetted to microplate wells and absorbance was measured at 517 nm. One hour after addition of 10 μL of the sample, the decrease in the absorbance was measured. The results were expressed as the percentage of DPPH radical inhibition (% inhibition).

The extracts were diluted 200-fold before performing the ORAC method. The method used was previously described by Burčul et al. [26]. In brief, the mixture of 150 μL of fluorescein and 25 μL of the sample (or Trolox for standard or puffer for blank) was pipetted to microplate wells and thermostated at 37 °C for 30 min. Following the addition of 25 μL of 2,2′-Azobis(2-amidinopropane) dihydrochloride (AAPH), measurements were made every minute for 80 min at excitation and emission wavelengths of 485 and 520 nm. ORAC results were expressed in µmole TE/L. 

The TPC and antioxidant assays were performed in triplicate. The absorbance of the extracts’ color was subtracted before the calculations.

The extract with the highest antioxidant activity was selected for chitosan/PLA bilayer film development.

### 2.3. Development of Chitosan/Polylactic Acid Bilayer Films

#### 2.3.1. Preparation of Bilayer Solutions

For this study, the solutions listed in Table 1 were prepared. Primary (low-molecular-weight chitosan (50 to 190 kDa), poly (D-glucosamine); Sigma-Aldrich, St. Louis, MO, USA) and quaternary (Chitosan Quaternary Ammonium Salt; CD Bioparticles, Shirley, NY, USA) chitosan solutions of 1% and 2% (*w/v*) were prepared by adding deionized water to chitosan powder with a few drops of absolute acetic acid (≥99.8%; Sigma-Aldrich, St. Louis, MO, USA) to dissolve the powder. The solutions were stirred overnight, and the pH was adjusted to 4.0 with acetic acid. Sodium tripolyphosphate (TPP; Sigma-Aldrich, St. Louis, MO, USA) was suspended in deionized water and stirred overnight to prepare a 0.2% (*w/v*) solution. The main purpose of setting pH = 4 was to ensure the same conditions for both chitosans. It should be taken into account that there are still 20% (the degree of substitution is 80%) of the primary groups in quaternary chitosan, which could be protonated in an acidic environment.

Before preparation of the chitosan particles, the freeze-dried *P. pavonica* June extract was dissolved in 50% ethanol (99.8%, GC; Sigma-Aldrich, St. Louis, MO, USA) to prepare a solution with a concentration of 10 mg/mL. The chitosan particles with the captured extract were then prepared using the ionic gelation technique. The prepared TPP and *P. pavonica* extract solutions were simultaneously added to a fixed volume of 1% (*w/v*) chitosan solution (primary and quaternary) to obtain a 5:1 weight ratio between chitosan and TPP, which was chosen according to our previous research [27]. The particles formed spontaneously under continuous stirring for 1 h at room temperature. The final pH of the chitosan particle dispersions with PPAV was adjusted to 4.0 with acetic acid under slow mixing.

#### 2.3.2. Application of Chitosan Solutions and Particles to PLA Films

The prepared chitosan solutions and chitosan particle dispersions were applied to the PLA films (Optimont^®^ PLA-Folie, Bleher Folientehnik GmbH, Ditzingen, Germany), which were cleaned and air-dried before application. The layers were applied directly to the surfaces of PLA in a roll-to-roll printing process using a printing table and a magnetic roller (Johannes Zimmer, Kufstein, Austria) at rolling speed level 3 and magnetic strength level 1. All previously prepared solutions were stirred before application and applied to the film surfaces in a fixed volume (primary and quaternary chitosan: 4 mL, chitosan particles with embedded extracts: 4 mL). Both layers were, thus, prepared under the same conditions and air-dried after each individual layer. The first layer consisted of 2% primary or 2% quaternary chitosan and the second layer consisted of chitosan particle dispersions (CHPs or QCHPs) with captured *P. pavonica* June extract. The description of the samples and their names are listed in Table 2.

#### 2.3.3. Physical and Chemical Properties of Chitosan/PLA Bilayer Films

The zeta potential (ZP) and hydrodynamic diameter (HD) of the prepared particle dispersions were determined using the particle size analyzer (Litesizer 500, Anton Paar, Graz, Austria) at 25 °C. ZP was measured by electrophoretic light scattering (ELS), which measures the velocity of particles in the presence of an electric field. HD was measured by dynamic light scattering (DLS). The speed of this motion depends on the size of the particles; smaller particles move faster than larger ones. Before analysis, the dispersion was stirred and, if necessary, adjusted to pH 4 with acetic acid. To perform the measurements, the diluted sample was placed in an Omega cuvette for ZP and size. Data were collected using Kalliope software (Anton Paar, Graz, Austria).

Contact angles were measured using a goniometer (Data Physics, Fidelstadt, Germany) to estimate the surface wettability of layered molding compounds. Milli-Q water (5 µL) was carefully placed on the test film surface. A goniometer with static contact angle (SCA) 20 software was used to determine SCA at room temperature. Analyses were performed in triplicates.

The chemical composition of the chitosan/PLA bilayer films was analyzed using the X-ray photoelectron spectroscopy (XPS) instrument model TFA-XPS (Physical Electronics, Munich, Germany). The spectrometer was equipped with a hemispherical electron analyzer and a monochromatic X-ray source with Al Kα1.2 radiation with a photon energy of 1486.6 eV. The excitation area of the sample was 400 µm^2^. The emitted photoelectrons were measured at a departure angle of 45°. During the XPS measurements, an electron gun was used to neutralize the surface charge. The survey spectra were measured at a transit energy of 187 eV and an energy step of 0.4 eV. MultiPak v8.1c software (Physical Electronics, Munich, Germany) was used to analyze the measured spectra.

Chitosan/PLA bilayer films were examined with a scanning electron microscope (SEM) using the JSM-IT800 instrument (Jeol, Tokyo, Japan). The PLA films were cut and glued to a double-sided conductive carbon tape, placed on a holder, and sprayed with gold to ensure conductivity and prevent charging effects. The samples were examined with an accelerating voltage of 5 kV and a variable working distance at comparable magnification. Images were acquired using a secondary electron detector.

### 2.4. Antioxidant Potential of Chitosan/PLA Bilayer Films

The antioxidant activity of the chitosan/PLA bilayer films was tested using 2,2′-azino-bis (3-ethylbenzothiazoline-6-sulfonic acid (ABTS) reagent. The assay is based on the spectrophotometric determination (UV-VIS) of the decolorization of the reagent in the presence of an antioxidant. The ABTS reagent (7 mM; Sigma-Aldrich, St. Louis, MO, USA) was prepared in 2.45 mM potassium persulfate (Sigma-Aldrich, St. Louis, MO, USA) and diluted with phosphate-buffered saline (PBS; Gibco, Life Technologies, Grand Island, NY, USA). Absorbance was measured at 734 nm and 25 °C at time points of 0 min, 15 min, and 60 min after addition of 0.1 g of film sample to 3.9 mL of ABTS solution. The solution was shaken during the extraction. The results are given as percentage of inhibition [18].

### 2.5. Statistical Analysis

Results are expressed as mean ± standard deviation. The statistically significant difference between *P. pavonica* extracts’ TPC and antioxidant activity over the months was determined by analysis of variance (one-way ANOVA), followed by a least significant difference test at the 95% confidence level [28]. Analyses were performed using Statgraphics Centurion-Ver.16.1.11 (StatPoint Technologies, Inc., Warrenton, VA, USA).

## 3. Results and Discussion

*Padina pavonica* was harvested from May to September during the period when its thallus grows in the Adriatic Sea. In particular, *P. pavonica*’s thallus detaches every winter and regrows in spring. After September and during the winter, this seaweed is in the form of rhizoids, filamentous thalli, or sporelings until spring comes and the conditions are suitable for its full regrowth [29]. Thus, its seasonal growth is from May till September. For this reason, we aimed to investigate the difference during thallus growth to see if there are significant changes in chemical profile and antioxidant activity during this algae’s seasonal growth. Knowing the perfect harvesting time can contribute to the knowledge needed for the possible cultivation or farming of this species and its potential exploitation.

### 3.1. Compound Analyses of P. pavonica Extracts

**Figure 2:**
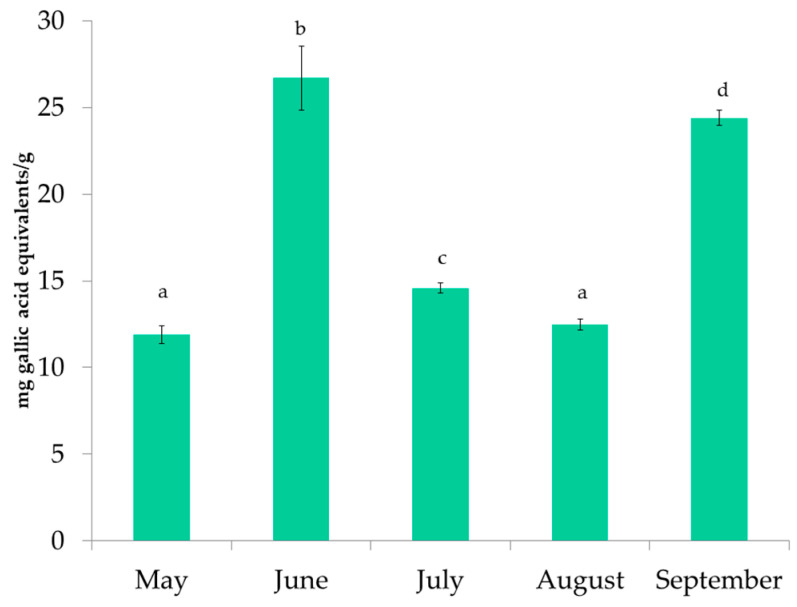
Total phenolic content of *P. pavonica* extracts. a–d different letters denote statistically significant difference.

The results of the TPC for *P. pavonica* harvested from May to September are shown in Figure 2. The highest TPC value was determined for the extract of *P. pavonica* harvested in June. Overall, the results varied between 11.88 ± 0.51 and 26.69 ± 1.86 mg GAE/g. The lowest TPC was found for the May sample. There are many factors that can affect the TPC in seaweeds. TPC can vary due to seasonal variations in salinity, sea temperature and light intensity, different geographic locations, and biological factors, such as algal life cycle, size, age, and the presence of predators [6]. To avoid the effects of geographic location, the seaweed samples in this study were collected from the same location and depth each month. The sea temperature and TPC results showed no correlation. The results of this study are in accordance with Bernardini et al. [30], who reported a TPC of 27.0 mg GAE/g in *P. pavonica* extract. In addition, Sofiana et al. [31] reported a TPC value of 20.34 mg GAE/g in the ethanolic extract of *P. pavonica*.

**Figure 3:**
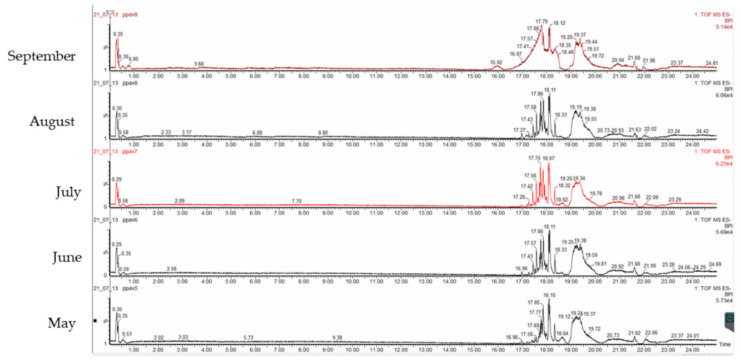
Chromatograms of the UPLC-PDA-ESI-QTOF analyses of *P. pavonica*.

*Padina pavonica* extracts were subjected to quali-quantitative analysis of polar compounds using LC-ESI-QTOF-MS in negative ion mode. The chromatograms of the basic peaks are shown in Figure 3. The results are listed in Table 3, along with their retention time, score (%), molecular formulae, observed and theoretical *m/z*, and error (ppm). Forty-seven compounds were tentatively identified. For all compounds, the error was lower than 5 ppm and the score was higher than 90%. All compounds were identified considering previous research [5] and the PubChem database. The amount of each compound was calculated based on the peak areas and expressed as a percentage.

Overall, most of the compounds detected were fatty acids. Oleic acid (C18:1*n-9*) was the predominant compound detected in *P. pavonica* extracts, with a content over 11% in all harvesting months. The highest content of oleic acid was detected in May, followed by June samples. Palmitic (C16:0) and palmitoleic (C16:1*n-7*) acids were also found in high amounts. Two ω-3 fatty acids, stearidonic acid (C18:4*n-3*) and eicosapentaenoic acid (EPA) (C20:5*n-3*), were also detected. Low molecular weight phenolic compounds were not identified. However, this does not exclude the presence of phenolics in the extracts. The reason could be the presence of high molecular weight phlorotannins in the extracts, which cannot be ionized and determined by HPLC-ESI-TOF-MS [5]. However, in our previous study, we identified phenolic acids (protocatechuic acid, *p*-hydroxybenzoic acid, *p*-coumaric acid, *t*-ferulic acid, and *o*-coumaric acid) in *P. pavonica* extracts using a different methodology [32]. Palmitic acid, palmitic acid ethyl ester, phytol, and phthalic acid were previously identified as major compounds in *P. pavonica* [33]. In addition, the same authors identified five phenolic compounds (kaempferol, ferulic acid, naringenin, delphinidin-3-oglucoside, and ellagic acid) in *P. pavonica* extracts [34]. Uslu et al. [35] identified myristic, palmitic, heptadecanoic, stearic, palmitoleic, and oleic acids in *P. pavonica*. The major fatty acids were margaric, palmitic, and oleic acids accounting for 39.55, 29.84, and 6.49% of the fatty acid composition, respectively. El Shoubaky and El Rahman Salem [36] identified palmitic acid (28.10%) and oleic acid (19.80%) as major fatty acids in the fatty acid composition of *P. pavonica*.

### 3.2. Antioxidant Activity of P. pavonica Extracts

**Figure 4:**
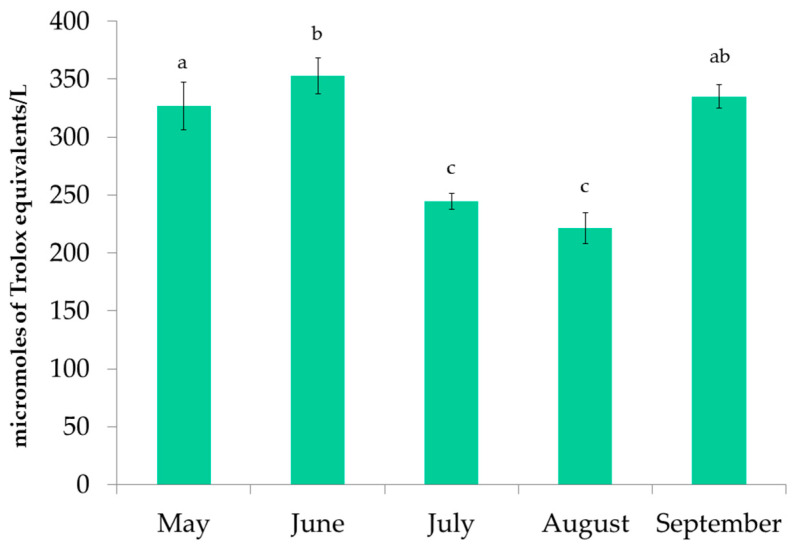
FRAP results for extracts of *P. pavonica* harvested from May to September. a–c different letters denote statistically significant difference.

**Figure 5:**
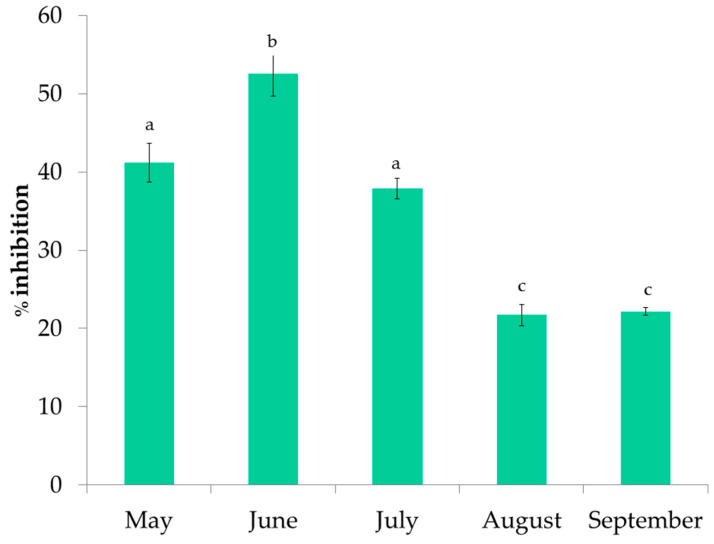
DPPH inhibition results for extracts of *P. pavonica* harvested from May to September. a–c different letters denote statistically significant difference.

**Figure 6:**
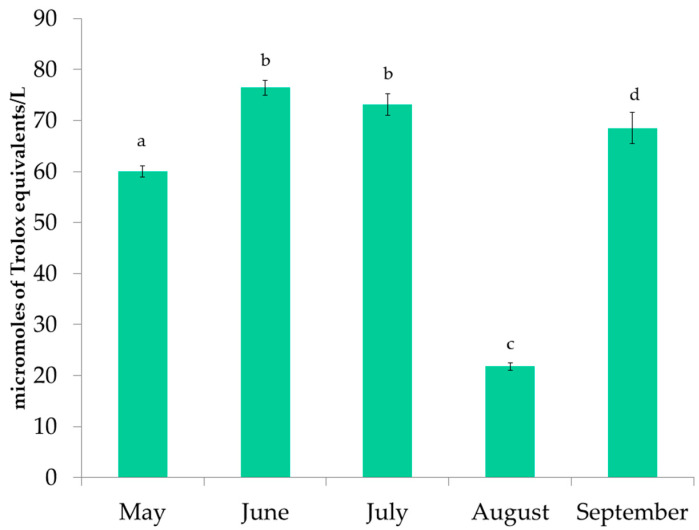
ORAC results for extracts of *P. pavonica* harvested from May to September. a–d different letters denote statistically significant difference.

The antioxidant activity of *P. pavonica* extracts was measured by the FRAP, DPPH, and ORAC methods. The results of FRAP are shown in Figure 4. The values ranged from 221.54 ± 13.41 to 352.82 ± 15.41 µmole TE/L. The highest reducing activity was observed in the June samples, followed by the September samples. In addition, the extracts from June had the highest DPPH inhibition (Figure 5) and the highest ORAC results (Figure 6). DPPH radical inhibition varied from 21.70 ± 1.34% to 52.51 ± 2.81%. The lowest inhibition was observed for the August sample, which was more than two times lower than the highest inhibition result. Prior to ORAC analyses, the extracts were diluted 200-fold. ORAC values ranged from 21.81 ± 0.71 to 76.45 ± 1.47 µmole TE/L. The lowest ORAC value was also found in the August sample.

Al-Enazi et al. [4] determined higher inhibition of DPPH radical (77.60%) for ethanolic extracts of *P. pavonica* harvested from the Red Sea (sampling season was not reported). Generalić Mekinić et al. [32] investigated the antioxidant activity of *P. pavonica* harvested from the Adriatic Sea in August 2020 but at a different location. Extracts were prepared in water and ethanol using ultrasound-assisted extraction. The highest FRAP (231 µmole TE) and ORAC (55.8 µmole TE) results were reported for the ethanolic extract. These results are comparable to the results of this study for the August samples. However, in this study, higher antioxidant activity measured by FRAP and ORAC was recorded for all months except for August. Kosanić et al. [37] investigated the antioxidant, antimicrobial and anticancer potential of *P. pavonica*, *Dictyota dichotoma*, and *Sargassum vulgare* acetone extracts. *Padina pavonica* extracts showed higher DPPH radical scavenging activity (IC_50_ of 691.56 µg/L) than other seaweed species.

### 3.3. Development of Chitosan/PLA Bilayer Films

Hydrodynamic diameter and zeta potential for all solutions used for PLA film layers are given in Table 4.

The average particle size for the CHPs was 358 nm and, for the QCHPs, 240 nm, whereas, for both chitosan particles with embedded *P. pavonica* extract (the June extract was selected for its highest antioxidant activity), the average particle size was 4873 and 6928 nm, respectively. The increase in particle size was evident because the incorporation of the active compounds from the extracts into or onto the surface of the chitosan particles increased the hydrodynamic diameter from colloidal size to micron scale. The increase in particle size confirms the successful incorporation of the extracts and the binding of the active compounds to the interior and/or surface of the chitosan particles [18,19].

According to the literature, dispersions/suspensions with a zeta potential greater than 30 mV are defined as stable suspensions with minimal sedimentation tendency. Thus, both chitosan’s dispersions are stable. No particle formulation with incorporated *P. pavonica* extract exceeded the zeta potential limit of 30 mV. The lowest zeta potential value was found in the CHP’sPPAV formulation at 18.8 mV. This could indicate that the extract is also bound to the surface of the chitosan particles or that the phenolic compounds interact with the amino groups, reducing the amount of available (free) amino groups and, thus, lowering the positive zeta potential [18,19].

The measured contact angles of the functionalized films are shown in Table 5. The results are shown as average angle and the percentage of angle reduction compared to the reference, an untreated PLA film.

The reference PLA film had an average value of 77.56°, proving the hydrophilicity of the film, but with a rather high contact angle. The same is true for the chitosan layers. Functionalization of the films with chitosan and *P. pavonica* extract layers (in particle suspensions) decreased the contact angle in all samples. In all cases, the contact angle was reduced. The reduction was 46%, 58%, 68%, and 58% for P3, P4, P9, and P10, respectively.

For all two-layered functionalized films, the contact angle decreased compared to the PLA reference, regardless of the type of layer. The application of the developed layers on the surface of the PLA films improved hydrophilicity, which is of outstanding added value for practical applications in the food industry, while improving the transparency of water droplets and eliminating the fogging effect.

The XPS composition results for the layers of *P. pavonica* extract and chitosan applied onto PLA film are shown in Table 6. Compared to the unlayered PLA film, a slightly lower oxygen content (containing: 66.4%, 32.9%, and 0.7% of carbon, oxygen, and silicon, respectively) and an additional presence of nitrogen and several microelements at low concentrations, such as Na, Mg, Si, P, Cl, Ca, S, and K, were observed. Nitrogen can be correlated with the presence of chitosan polymers, P to particles, while other microelements should belong to the *P. pavonica* extract, clearly confirming the presence of the layers on the PLA surface. One possible reason that P was not detected in all samples, even though all samples are layered with nanoparticles, could be the nonuniformity of the layers, as seen at SEM, and the fact that XPS analysis is limited due to the very thin surface layer of about 50–100 nm (which does not necessarily mean that P is available; as an ion, it can penetrate deeper). After desorption, some microelements are no longer observed; however, other microelements remain on the surface. The inhomogeneity of the layers leads to illogical concentration variations, i.e., for some elements, the concentration increases after desorption.

Potrč et al. [18] and Zemljič et al. [19] used a very similar deposition of formulations on films and found that the low desorption was due to a sequential deposition strategy: a 2% solution of primary chitosan was used as the first layer, followed by a dispersion of chitosan particles with the active ingredient—the extract—as the second layer. The same deposition strategy was used in this study. This could increase the stability, as the macromolecular chitosan solution serves to improve the adhesion of the chitosan nanoparticles with the incorporated extract, thus increasing the stability of the phenolic compounds at the surface of the film [18,19].

**Figure 7:**
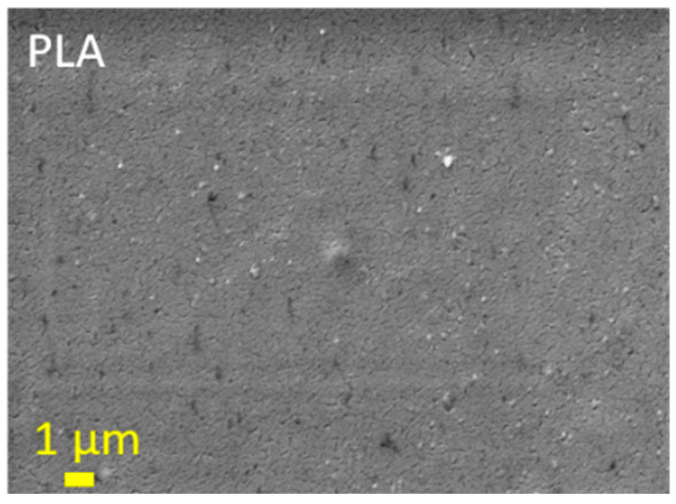
SEM image of uncoated PLA film that was used as reference.

**Figure 8:**
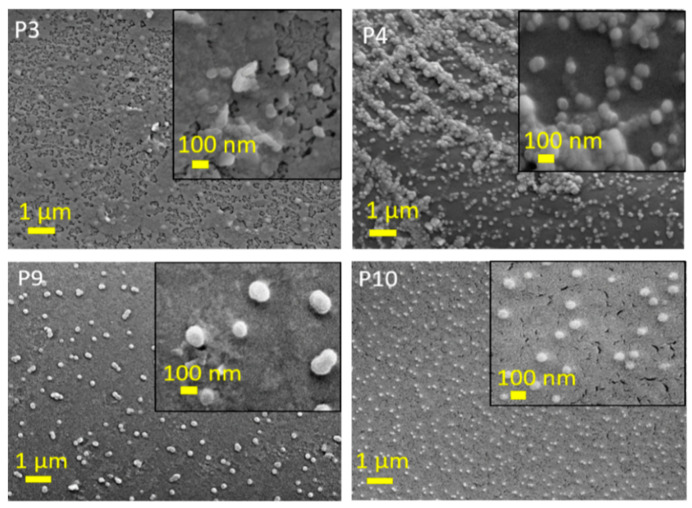
SEM images for PLA films layered first with 2% CH or QCH, followed by second layer in form of micro/nanoparticles of CH or QCH with embedded *P. pavonica* extract.

The results of the SEM analyses are shown in Figure 7 and Figure 8. To further investigate the morphology of the PLA films after the two-layers application, SEM was used to visualize the coverage of the films. The untreated PLA film shows a typical flat surface with some imperfections, possibly due to impurities, as also shown by XPS (Si). The layered samples show changes in morphology. Samples P3 and P4, containing a top layer with CH and QCHPs with embedded PPAV extract, show, in the case of P3, that the particles are embedded in the applied first layer of macromolecular chitosan solutions and particles forming dendrite clusters are clearly visible, the size of the particles being also below 100 nm. In the applications with the 2% QCH first layer, i.e., samples P9 and P10, similar morphologies are observed as in the samples with the first layer of 2% CH. However, due to the presence of fewer agglomerates and evenly distributed particles, some differences can be observed. This is especially true for samples P9 and P10, which contain particles with entrapped PPAV extract.

The results of the analyses of SEM are shown in Figure 7 and Figure 8. To further investigate the morphology of the PLA films after bilayer application, SEM was used to visualize the coverage of the films. The original PLA film shows a typical flat surface with some impurities, possibly due to contamination, as also shown by XPS analysis (Si). The layered samples show a change in morphology. Samples P3 and P4, containing a first layer of primary chitosan and a top layer with CH and QCHPs with embedded PPAV extract, show that nanoparticles are embedded in the applied first layer or particles forming dendrite assemblies are clearly visible, with the size of the particles also below 100 nm. In samples P9 and P10, where the first layer consists of 2% QCH, similar morphologies are observed as in the samples with the first layer composed of 2% CH. Nevertheless, some differences can be seen due to the presence of fewer agglomerates and uniformly distributed particles.

### 3.4. Antioxidant Potential of Chitosan/PLA Bilayer Films

**Figure 9:**
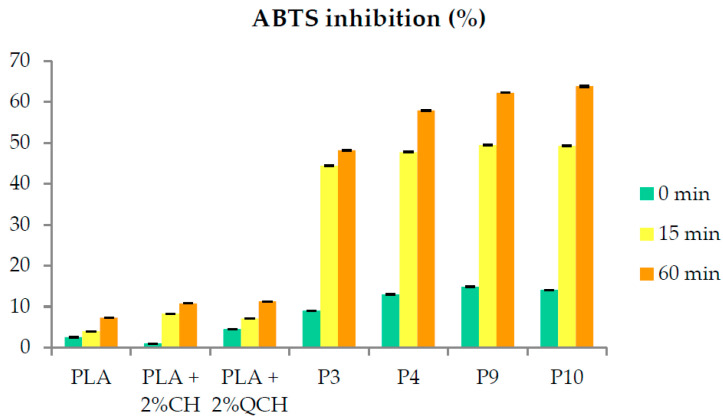
ABTS inhibition after 0, 15, and 60 min for all films.

The antioxidant activity of the developed films was investigated using the ABTS method to determine how the layers on the surface of the PLA films affect the inhibition of the oxidation process that commonly occurs during food storage. The ABTS inhibition results of the PLA film samples after 0, 15, and 60 min are shown in Figure 9.

The results show that the lowest antioxidant activity was obtained for the PLA films and the PLA films layered with chitosans alone. Chitosans alone as layers on the films did not show good antioxidant activity. It can be seen that, in both chitosans, the first layers generally showed antioxidant activity below 10%. For the two-layer PLA films based on chitosans and *P. pavonica* extract, the inhibition immediately after addition of the ABTS solution was low and ranged from 8.99 to 14.84%. After 15 min, inhibition was generally 47% and was highest for samples P9 and P10, where it reached 49.5%. However, after 60 min, the inhibition for the film samples with *P. pavonica* extract was 63% for P9 and P10. The lowest inhibition was observed for P3, with 48%, and for P4, with almost 58%. It is evident that the antioxidant capacity of *P. pavonica* extracts was preserved in these film formulations.

There are several studies that investigated film-forming properties and application of seaweed polysaccharides for the development of edible coatings and films [38,39]. However, only few studies have investigated the effectiveness of seaweed extracts for the coatings or films development. Albertos et al. [40] developed edible chitosan films with added seaweeds (*Himanthalia elongata* and *Palmaria palmata*) and seaweed extracts. The TPC of the extracts ranged from 46.72 to 206.69 mg GAE/g sample and extracts with higher TPC exhibited higher antioxidant activity. The antioxidant activity of the formulated edible films was consistent with the antioxidant activity of the extracts. The films were then tested on rainbow trout (*Oncorhynchus mykiss*) burgers, where the authors observed a reduction in lipid oxidation and microbial growth, as well as an improvement in the antioxidant capacity of the burgers during the storage. Andrade et al. [41] developed film based on whey protein with incorporated extract of *Fucus vesiculosus*. The TPC of the extract was 45.21 ± 0.21 mg phloroglucinol equivalents/g extract and the inhibition of DPPH radical was 78.26 ± 0.21%. The developed film inhibited lipid oxidation of vacuum-packed chicken breasts for 25 days under refrigerated conditions.

## 4. Conclusions

The most dominant compounds of *P. pavonica* extracts, determined by HPLC-ESI-TOF-MS, were fatty acids (oleic, palmitic, and palmitoleic). In addition, ω-3 fatty acids, stearidonic and EPA, were identified. *Padina pavonica* harvested in June showed the highest TPC and antioxidant activity. This extract was selected for the development of chitosan/PLA bilayer films. This is the first report of testing such a formulation. A macromolecular solution of chitosan (primary and quaternary chitosan) was applied as the first layer on PLA films, and a second layer consisted of a suspension of chitosan particles with entrapped PPAV extract. XPS spectra confirmed that both layers, first as chitosan macromolecular solutions and second as PPAV extract embedded into chitosan particles, were successfully deposited on the film surface, with some desorption. In addition, the hydrophilicity of the films was reduced, which is very important for ensuring food safety and quality due to the anti-fog effect. An increase in antioxidant activity in the range of 48–65% was observed for all functionalized films. The developed films exhibited anti-fogging and antioxidant properties, confirming the development of the active concept and the potential use of these films for food packaging solutions.

## Figures and Tables

**Table 1:** Sample description of prepared solutions.

Solutions	Abbreviation
1% primary chitosan	CH
1% quaternary chitosan	QCH
2% primary chitosan	2%CH
2% quaternary chitosan	2%QCH
Primary Chitosan particles	CHP’s
Quaternary Chitosan particles	QCHP’s
*P. pavonica* June extract	PPAV
Primary chitosan particles with captured extract	CHP’sPPAV
Quaternary Chitosan particles with captured extract	QCHP’sPPAV
Sodium tripolyphosphate	TPP

**Table 2:** PLA film samples description.

Description of the Samples	Sample Name
References	
PLA with no layers	PLA
PLA applicated with 2% CH	PLA + 2%CH
PLA applicated with 2% QCH	PLA + 2%QCH
Samples with primary chitosan as first layer	
PLA applicated with 2% CH and CHP’sPPAV	P3
PLA applicated with 2% CH and QCHP’sPPAV	P4
Samples with quaternary chitosan as first layer	
PLA applicated with 2% QCH and CHP’sPPAV	P9
PLA applicated with 2% QCH and QCHP’sPPAV	P10

**Table 3:** The list of compounds detected in *P. pavonica* extracts analyzed by UPLC-PDA-ESI-QTOF.

No.	RT (min)	Observed *m/z*	Theorical *m/z*	Error (ppm)	Score (%)	Molecular Formulae	Tentative Compound	May (%)	June (%)	July (%)	August (%)	September (%)
1	0.27	343.0367	343.0368	−0.3	94.07	C_20_ H_4_ N_6_ O	1a,9b-Dihydrophenanthro [9,10-b]oxirene-2,3,4,7,8,9-hexacarbonitrile	7.61	6.45	4.78	6.42	6.63
2	0.29	201.0239	201.0247	−4.0	98.89	C_4_ H_10_ O_9_	2-(1,2,2,2-Tetrahydroxyethoxy)ethane-1,1,1,2-tetrol	7.62	7.13	5.76	6.72	7.05
3	0.34	141.0157	141.0161	−2.8	91.01	C_2_ H_2_ N_6_ O_2_	Diazidoacetic acid	0.82	1.27	1.30	0.80	1.05
4	0.35	181.0709	181.0712	−1.7	100	C_6_ H_14_ O_6_	D-Sorbitol	1.56	1.14	1.35	1.12	2.44
5	0.39	317.0516	317.0509	2.2	90.44	C_12_ H_14_ O_10_	D-glucaric acid derivate	0.50	0.45	0.55	0.52	1.62
6	16.60	343.2122	343.2121	0.3	95.77	C_18_ H_32_ O_6_	10,11-Dihydroxy-9,12-dioxooctadecanoic acid	0.15	0.14	0.12	0.13	0.13
7	16.84	487.3426	487.3423	0.6	96.96	C_30_ H_48_ O_5_	Esculentic acid	0.36	0.00	0.00	0.00	0.00
8	16.96	275.2012	275.2011	0.4	100	C_18_ H_28_ O_2_	Stearidonic acid (C18:4*n-3*) isomer a	0.08	0.08	0.04	0.16	0.41
9	17.10	309.2056	309.2066	−3.2	96.09	C_18_ H_30_ O_4_	6,9-Octadecadienedioic acid	0.20	0.33	0.17	0.26	0.32
10	17.16	285.2066	285.2066	0.0	90.36	C_16_ H_30_ O_4_	Hexadecanedioic acid	0.32	0.14	0.07	0.14	0.21
11	17.17	277.2168	277.2168	0.0	90.14	C_18_ H_30_ O_2_	gamma-Linolenic acid isomer a (C18:3*n-6*)	0.12	0.11	0.08	0.30	0.53
12	17.19	295.2276	295.2273	1.0	100	C_18_ H_32_ O_3_	9,10-Epoxyoctadecenoic acid isomer a (vernolic acid)	0.45	0.20	0.47	0.29	0.14
13	17.22	429.30090	429.3005	0.9	91.64	C_27_ H_42_ O_4_	24-Keto-1,25-dihydroxyvitamin D3 isomer a	n.d.*	0.02	n.d.	0.01	0.03
14	17.27	247.1712	247.1698	5.7	100	C_16_ H_24_ O_2_	2,4,6-Triisopropyl benzoic acid	0.17	0.60	1.09	0.77	0.48
15	17.30	297.2426	297.2430	−1.3	98.84	C_18_ H_34_ O_3_	10-Oxooctadecanoic acid isomer a	1.14	0.43	n.d.	0.34	0.29
16	17.34	287.2212	287.2222	−3.5	90.62	C_16_ H_32_ O_4_	10,16-Dihydroxyhexadecanoic acid isomer a	0.07	0.09	0.10	0.09	0.16
17	17.35	287.2211	287.2222	−3.8	90.73	C_16_ H_32_ O_4_	10,16-Dihydroxyhexadecanoic acid isomer b	0.03	0.16	0.67	0.58	1.40
18	17.37	199.16890	199.1698	−4.5	92.54	C_12_ H_24_ O_2_	Lauric acid	0.60	0.63	0.57	0.57	0.43
19	17.38	243.1952	243.1960	−3.3	90.78	C_14_ H_28_ O_3_	3-hydroxymyristic acid	0.68	0.32	0.37	0.34	0.40
20	17.42	293.2117	293.2117	0.0	80.96	C_18_ H_30_ O_3_	13-ketooctadecadienoic acid isomer a	0.33	0.22	0.57	0.29	0.12
21	17.43	293.2117	293.2117	0.0	87.56	C_18_ H_30_ O_3_	13-ketooctadecadienoic acid isomer b	0.46	1.93	2.47	1.95	3.15
22	17.44	295.2276	295.2273	1.0	100	C_18_ H_32_ O_3_	9,10-Epoxyoctadecenoic acid isomer b (vernolic acid)	1.00	0.34	n.d.	0.29	0.24
23	17.51	269.2110	269.2117	−2.6	98.63	C_16_ H_30_ O_3_	3-Oxohexadecanoic acid	1.29	1.05	1.11	1.12	2.11
24	17.51	225.1847	225.1855	−3.6	95.99	C_14_ H_26_ O_2_	Myristoleic acid	1.53	1.44	1.27	1.33	1.03
25	17.56	275.2007	275.2011	−1.5	36.37	C_18_ H_28_ O_2_	Stearidonic acid (C18:4*n-3*) isomer b	0.72	0.66	0.40	1.09	2.19
26	17.58	275.2010	275.2011	−0.4	93.59	C_18_ H_28_ O_2_	Stearidonic acid (C18:4*n-3*) isomer c	1.13	4.46	4.38	3.85	2.88
27	17.59	277.2159	277.2168	−3.2	91.36	C_18_ H_30_ O_2_	gamma-Linolenic acid isomer b (C18:3*n-6*)	0.01	0.08	0.03	0.07	0.04
28	17.60	213.18450	213.1855	−4.7	92.41	C_13_ H_26_ O_2_	Tridecanoic acid	0.82	0.75	0.62	0.72	0.54
29	17.61	257.2108	257.2117	−3.5	95.16	C_15_ H_30_ O_3_	11-Hydroxypentadecanoic acid	0.43	0.16	n.d.	0.17	0.22
30	17.63	251.2010	251.2011	−0.4	100	C_16_ H_28_ O_2_	7-cis,10-cis-hexadecadienoic acid	0.72	0.94	1.07	1.12	0.61
31	17.64	297.2429	297.2430	−0.3	97.33	C_18_ H_34_ O_3_	10-Oxooctadecanoic acid isomer b	0.88	0.42	n.d.	0.55	0.39
32	17.66	239.2004	239.2011	−2.9	98.8	C_15_ H_28_ O_2_	Myristoleic acid methyl ester	3.69	3.23	2.67	3.06	2.39
33	17.70	301.2156	301.2168	−4	98.12	C_20_ H_30_ O_2_	Eicosapentanoic acid isomer a (C20:5*n-3*)	0.96	3.26	3.49	3.39	2.36
34	17.75	277.2171	277.2168	1.1	50.48	C_18_ H_30_ O_2_	gamma-Linolenic acid isomer c (C18:3*n-6*)	2.75	5.28	6.90	5.60	5.16
35	17.77	227.2005	227.2011	−2.6	94.05	C_14_ H_28_ O_2_	Tetradecanoic acid (C14:0)	5.16	4.13	4.47	4.01	3.13
36	17.80	271.2266	271.2273	−2.6	97.75	C_16_H_32_O_3_	Hydroxy-palmitic acid	3.43	1.40	1.76	1.46	1.91
37	17.85	253.2159	253.2168	−3.6	99.61	C_16_ H_30_ O_2_	Palmitoleic acid (C16:1*n-7*)	10.10	9.25	9.71	9.28	8.84
38	17.91	279.2319	279.2324	−1.8	98.14	C_18_ H_32_ O_2_	Octadeca-10,12-dienoic acid (C18:2*n-6*) isomer a	1.49	1.68	2.55	2.08	2.32
39	17.93	241.2168	241.2168	0.0	100	C_15_ H_30_ O_2_	Pentadecanoic acid (C15:0)	2.89	2.65	2.39	2.57	2.49
40	17.97	279.2324	279.2324	0.0	91.12	C_18_ H_32_ O_2_	Octadeca-10,12-dienoic acid (C18:2*n-6*) isomer b	1.93	1.84	1.91	1.73	1.58
41	18.00	267.2329	267.2324	1.9	100	C_17_ H_32_ O_2_	9-Heptadecenoic acid (C17:1*n-8*)	2.81	2.88	2.47	2.70	2.86
42	18.07	255.2318	255.2324	−2.4	99.95	C_16_ H_32_ O_2_	Hexadecanoic acid (palmitic acid) isomer a (C16:0)	0.24	0.31	0.39	0.35	0.45
43	18.08	255.2318	255.2324	−2.4	99.44	C_16_ H_32_ O_2_	Hexadecanoic acid (palmitic acid) isomer b (C16:0)	9.40	8.43	9.19	8.67	8.11
44	18.10	281.2472	281.2481	−3.2	99.96	C_18_ H_34_ O_2_	Oleic acid (C18:1*n-9*)	13.85	12.59	11.68	11.81	11.48
45	18.21	269.2474	269.2481	−2.6	97.24	C_17_ H_34_ O_2_	Heptadecanoic acid (C17:0)	3.78	3.64	3.27	3.59	3.33
46	18.33	283.2629	283.2637	−2.8	99.97	C_18_ H_36_ O_2_	Octadecanoic acid (stearic acid) C18:0	4.33	3.76	3.74	3.65	3.40
47	18.54	311.2944	311.295	−1.9	92.83	C_20_ H_40_ O_2_	Arachidic acid	0.55	0.46	0.53	0.49	0.49

* n.d.—not detected.

**Table 4:** Hydrodynamic diameter (HD) and zeta potential (ZP) with standard deviation (SD) of different formulations.

Sample	HD (nm)	ZP ± SD (mV)	pH
CHP’sPPAV	4873	18.8 ± 0.4	4
QCHP’sPPAV	6928	23.6 ± 0.4	4
CHPs	358	36 ± 0.2	4
QCHPs	239.9	33 ± 0.3	4

**Table 5:** Contact angles for PLA, reference samples, and P3, P4, P9, and P10.

Samples	Average Angle (α/°)	Difference (%)
PLA	77.56 ± 1.61	/
PLA + 2%CH	80.39 ± 2.23	−3.66 ± 2.23
PLA + 2%QCH	78.69 ± 4.02	−1.47 ± 4.02
P3	41.38 ± 0.98	46.65 ± 0.98
P4	32.50 ± 0.25	58.10 ± 0.25
P9	24.93 ± 0.43	67.86 ± 0.43
P10	32.54 ± 1.91	58.18 ± 1.91

**Table 6:** XPS analysis of functionalized PLA films with *P. pavonica* extract before and after desorption (in %).

Sample	C	N	O	Na	Mg	Si	P	Cl	Ca	S	K
Before Desorption											
P3	65.8	1.2	30.3	0.3	1.2	0.3	0	0.8	0.2	0.3	0
P4	62.4	2.6	31.9	0.4	0.5	0.3	0.2	1.7	0.1	0	0
P9	67.3	2.2	26.3	0.3	0.8	1.5	0	1.3	0.2	0	0
P10	62.8	5.6	26.1	0.7	1	0.4	0	3.3	0	0.2	0
After Desorption											
P3	66.6	4.0	26.7	0.3	0	1.5	0.4	0.2	0	0.4	0
P4	65.0	1.7	32.5	0	0	0.4	0	0.2	0	0.2	0
P9	60.9	4.2	30.2	1.9	0	0.8	0	0.8	0.2	0.4	0.7
P10	66.1	4.4	26.7	0	0	1.3	0.3	0.5	0	0.6	0

## Data Availability

The data are available from the corresponding author.
